# Different patterns of human activities in nature during Covid-19 pandemic and African swine fever outbreak confirm direct impact on wildlife disruption

**DOI:** 10.1038/s41598-021-99862-0

**Published:** 2021-10-21

**Authors:** Jan Cukor, Rostislav Linda, Karolina Mahlerová, Zdeněk Vacek, Monika Faltusová, Petr Marada, František Havránek, Vlastimil Hart

**Affiliations:** 1 Faculty of Forestry and Wood Sciences, Czech University of Life Sciences Prague, Kamýcká 129, 165 00 Prague 6, Suchdol, Czech Republic; 2 Forestry and Game Management Research Institute, V.V.I, Strnady 136, 252 02 Jíloviště, Czech Republic; 3 Faculty of Environmental Sciences, Czech University of Life Sciences Prague, Kamýcká 129, 165 00 Prague 6, Suchdol, Czech Republic; 4 Faculty of AgriSciences, Mendel University in Brno, Zemědělská 1, 613 00 Brno, Czech Republic

**Keywords:** Ecology, Zoology, Ecology

## Abstract

Implementation of various restrictions to eradicate viral diseases has globally affected human activity and subsequently nature. But how can the altered routines of human activity (restrictions, lockdowns) affect wildlife behaviour? This study compared the differences between human and wildlife occurrences in the study forest area with acreage of 5430.6 ha in 2018 (African swine fever outbreak, complete entrance ban), 2019 (standard pattern) and 2020 (COVID-19 restrictions) during the breeding season. The number of visitors was lower by 64% in 2018 (non-respecting of the entry ban by forest visitors) compared to standard 2019, while in 2020, the number of visitors increased to 151%. In the COVID-19 period, distinct peaks in the number of visitors were observed between 8–11 AM and 4–7 PM. The peaks of wildlife activity were recorded between 4–7 AM and 9–12 PM. Animals avoided the localities that were visited by humans during the people-influenced time (24 h after people visit), which confirmed the direct negative impact of human activities on wildlife.

## Introduction

Land-use changes, including urbanization, have led to severe habitat fragmentation, degradation, and loss, so therefore humans and wildlife live in closer proximity^1^. Human-wildlife interactions affect the behaviour and movement of both parties^2^. Outdoor recreational activities disturb wildlife in terms of the energy expenditure, impact on animal behaviour and physical fitness, and cause circumventing an otherwise suitable habitat, synergistically resulting in changes in wildlife activity, feeding time, reproduction, and survival^3–5^. Therefore, tourism and recreation are considered a major threat to wilderness ecosystems^5,6^. The negative impact of tourism and recreation has been known for almost a century^7^. The rising impact of recreational activities on the environment goes hand in hand with the growing numbers of outdoor recreationists^5,8^, which is globally documented, especially in protected areas and urban forests^5,9,10^.

The development of nature tourism and recreation in forests is related to increasing interest in outdoor sports activities such as hiking, skiing, horseback riding, biking, berry and mushroom foraging, short-term camping, walking, and dog walking^5,7,11^. Other factors are the adequate accessibility of nature areas with well-developed road networks^5,12^. The impact of recreation and tourism changes in relation to landscape characteristics where the long-term impact on wildlife might be particularly high in the urban framework and is related to the local population size^13,14^. The number of visitors in nature are related to various rules of protection, especially in national parks and protected areas ^13,15,16^. Furthermore, political decisions such as different patterns of human activity in relation to the eradication of serious viral diseases that affect continents or are widespread globally, undoubtedly influenced the extent of nature tourism and recreation activities^17,18^.

Currently, the COVID-19 pandemic is the greatest health and economic challenge in modern history, which threatens millions of human lives and has devastating consequences on social and economic life worldwide^19–21^. The virus spread globally within about 2 months from its origin in Wuhan, China^22^, infecting people at an exponential rate, and leading to measures that are disrupting the global economy in an attempt to contain it ^23,24^. The available methods to mitigate the spread of the epidemic are standard control measures, such as social distancing (mitigating contacts by home office), hand hygiene, face mask use, isolation of confirmed cases, contact tracing, and quarantine^25,26^. Moreover, many countries around the world went into lockdown to control the spread of the virus^27^, with total shutdowns of whole large and small cities^28^. The reduction in human mobility on land and at sea including air transport is unprecedented in recent history^29,30^. Therefore, the general assumption is that the reduction of traffic and other human activities leads to improving the wildlife environment^31^, and reduces the stress to wildlife^19^. First estimations and unofficial observations from the beginning of the COVID-19 pandemic has indicated that many animal species are enjoying the unforeseen peace and quiet reflected in significant changes in the environment and the natural habitats^27^. The recent field data confirmed the positive effects on wildlife conservation, such as reduced stress on sensitive animals, increased species richness in less disturbed habitats, higher breeding success of aerial insectivorous birds, and reduced wildlife collisions with traffic^32,33^. In the first months of pandemic, the positive effect of the COVID-19 on wildlife was observed principally in national parks and protected areas where the dramatic declines in the number of visitors is easily measured^33,34^. However, there is limited knowledge on the impact of the COVID-19 pandemic on wildlife on a local scale.

Another serious viral disease impacting human behaviour and causing considerable socio-economic losses is African swine fever (ASF)^35,36^. The current outbreaks of the epidemic have been reported from 17 European and 12 Asian countries^37^. The impact of the ASF outbreak in China led to the reduction of pig industry by 9–34% in global production, which leads to increasing pork prices by 17–85%^38^. The spread of African swine fever is exacerbated by several factors such as the natural movement of wild boars and direct contact between individuals^37,39^. Forestry and human leisure activities can also affect the disease transmission by the disruption and subsequent movement of infected wild boar^40^. Therefore, one of the most effective measures is to forbid entrance into the areas with local outbreaks, as was done in 2018 the Czech Republic (described in the Methods section).

The last 3 years gave us the unique opportunity to compare different levels of outdoor activities according to legislative regulations, which influence the human behaviour in relation to aforementioned viral diseases. The Czech Republic was affected by African swine fever in 2018, with movement restriction enforced in the outbreak area. In 2019, ASF was eradicated and human movement returned to the standard regimen without any restrictions. In 2020, the COVID-19 pandemic emerged, which brought several mitigation measures including the closing of schools, recommending working from home, and finally, a full lockdown. Nevertheless, walks in nature, staying in the sunlight and other outdoor activities were highly recommended due to improving health status and immunity^41–43^. However, the direct impact of these measures on wildlife, especially in the breeding season (spring months), was not considered. Therefore, the objectives of this study were to: (1) evaluate the efficiency of the ban on entering the forest during the ASF outbreak in 2018; (2) compare the prohibition regimen with normal forest visiting in 2019; (3) describe altered patterns of human visits and wildlife occurrences in forests in the normal situation (2019) and during the COVID-19 pandemic (2020); and (4) determine the effect of the frequency of human presence on wildlife, all in the forest ecosystems close to urban centers.

## Results

**Figure 1:**
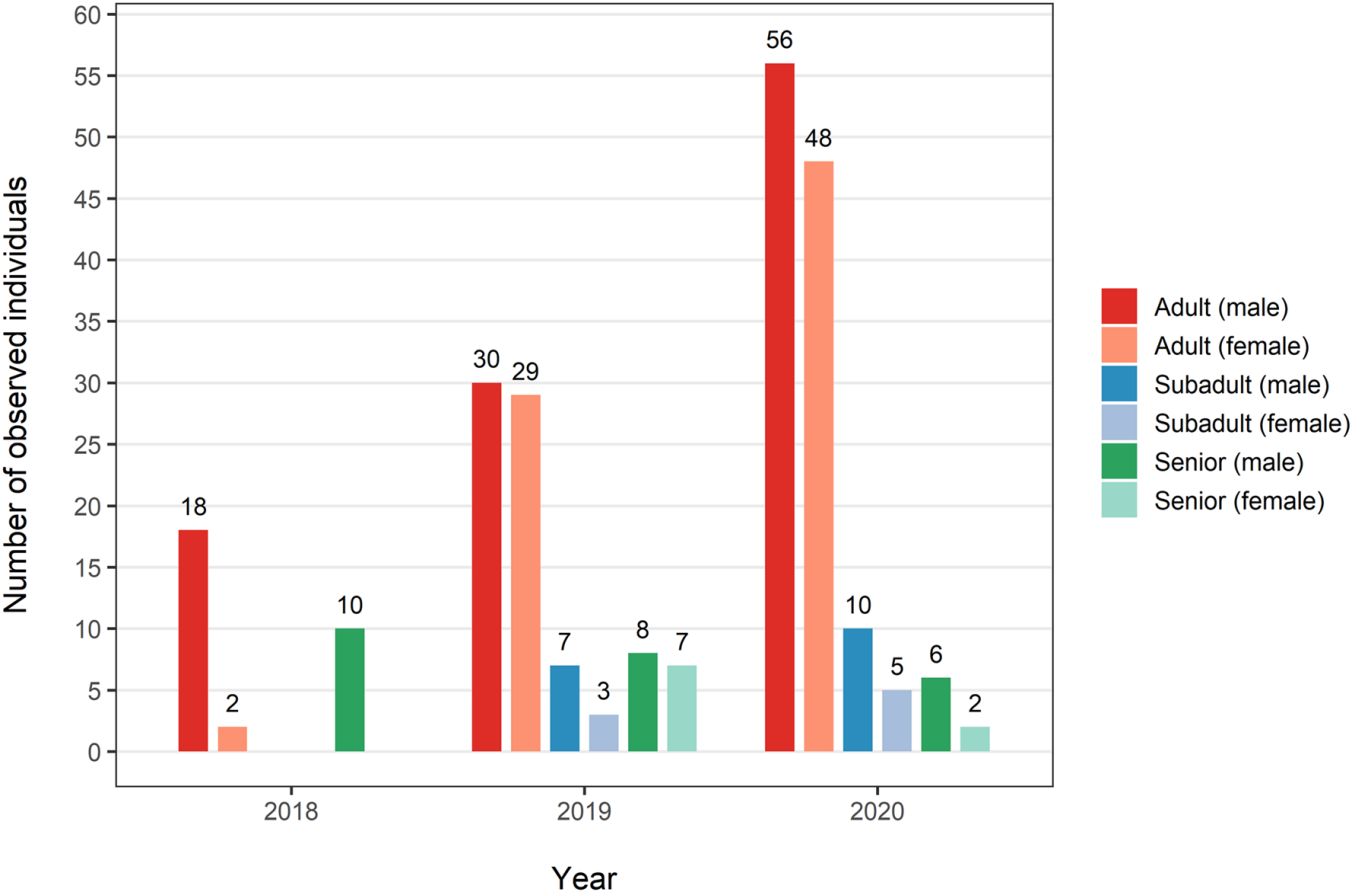
Numbers of records divided by age category and gender in studied months of selected years.

In total, we have recorded 241 people via camera traps from the selected study area (30 people in 2018, 84 in 2019, and 127 in 2020). Men and women accounted for 60.2% and 39.8% of the total number of visitors, respectively (93.3% and 6.7% in 2018, 53.6% and 46.4% in 2019, 56.7% and 43.3% in 2019). The numbers of records were divided to subadults, adults and seniors, and further by gender, as depicted in Fig. 1.

The positive trend of recorded adults (male and female together) was tested via linear regression and was statistically significant (*p* = 0.026). Among all records, 6 people were apparently working in the forest, 5 in 2018 and 1 in 2019.

The most people were recorded on June 25, 2020 (Thursday, 12 people), followed by May 24, 2020 (Sunday, 10 people); in 2019, the most people were recorded on May 7 (Tuesday, a day before the public holiday), May 9 (Thursday, a day after the public holiday), and June 29 (Saturday); in 2018, on May 26 (Saturday) and June 13 (Wednesday).

**Figure 2:**
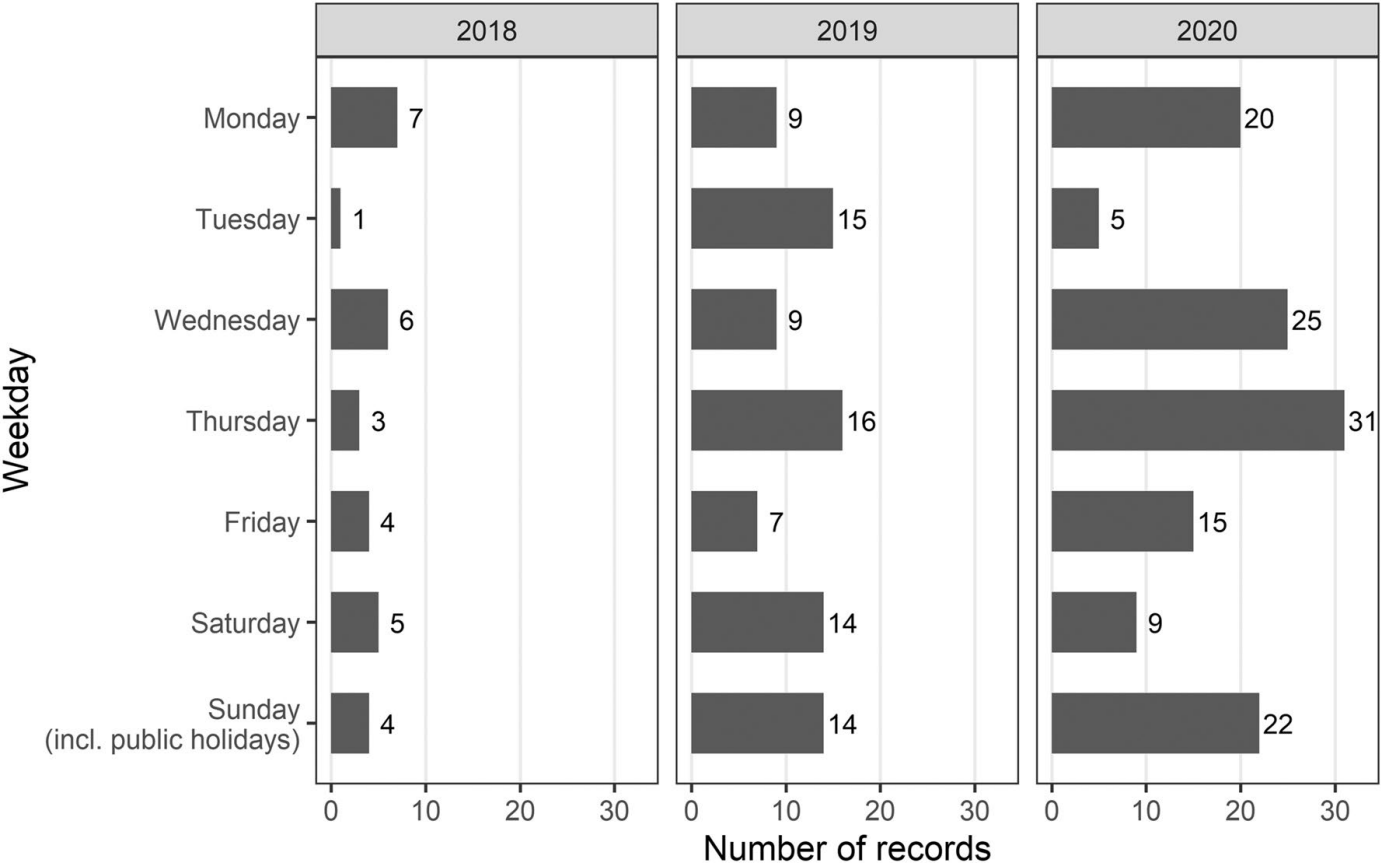
The distribution of human records on different weekdays.

Regarding the weekdays, the highest number of records was observed on Thursday (50 records, 20.7%), followed by Wednesday and Sunday, including public holidays (both 40 records, 16.6% each). Differing frequencies of human activities between the weekdays and weekends were observed. In 2018 we detected 107.1% of visits during the weekends compared to weekdays. In standard pattern (2019) was the difference most prominent during the weekends (125.0% of visitors compared to weekdays). In COVID-19 period (2020) was observed the opposite situation when during the weekends the camera traps recorded 80.7% of visitors compared to weekdays. The distribution of records during weekdays is depicted in Fig. 2.

The chi-square test for equality of probabilities for people detection on different weekdays showed significant differences between them (Chi-squared = 17.419, df = 6, *p* = 0.008). Thursday was found as the most frequent day in 2019 and 2020 (there are not enough records in 2018 to make a solid conclusion about the most frequent day), but there were significant differences found in the proportion of human records on different weekdays between 2019 and 2020 (Chi-squared = 19.301, df = 6, *p* = 0.004). The most prominent differences were observed in the case of Tuesday (which accounts for 17.9% in 2019, but only for 3.9% in 2020) and Saturday (16.7% in 2019, but only 7.1% in 2020).

**Figure 3:**
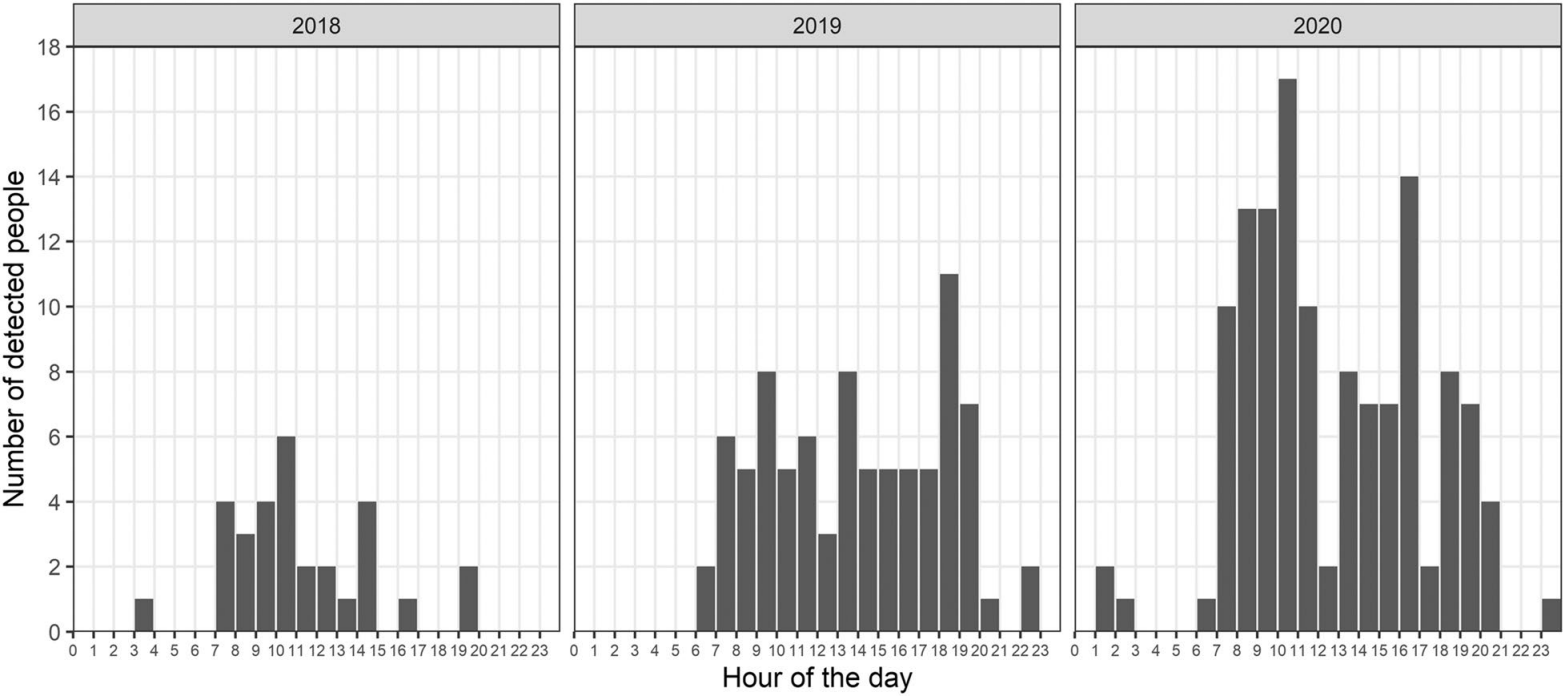
Hour histogram of people recorded on camera traps during the study period.

The overall analysis of the daytime hours showed that most of the records appeared in the morning hours; 46.5% of all individuals were recorded between 7 and 12 AM. During the day hours (from 4 AM to 8 PM), 229 out of total 241 people (95.0%) were observed. Most were observed between 10 and 11 AM (28 records) and between 9 and 10 AM (25 records). Relatively high counts were also observed in the afternoon, e.g. 20 records between 4 and 5 PM and 19 records between 6 and 7 PM. An hour histogram of detected people is depicted in Fig. 3.

We have found one revealing peak in the number of recorded people in 2018 (around 10 AM). The majority of the people in 2018 were observed between 7 AM and 3 PM (26 out of 30 records in 2018; 87%). Contrary to 2018, we found an “evening peak” in 2019 and in 2020. In 2019, we observed a relatively stable frequency between 7 AM and 8 PM with a few peaks around 9 AM, 1 PM and 6 PM, and one drop around lunchtime. In total, 79 out of 84 (94%) recorded people were observed between 7 AM and 8 PM in 2019. In 2020, we observed two main peaks in human records, at approximately 10 AM and 4 PM. In 2020, people were usually recorded between 7 AM and 9 PM (122 out of 127 cases, 96%).

**Figure 4:**
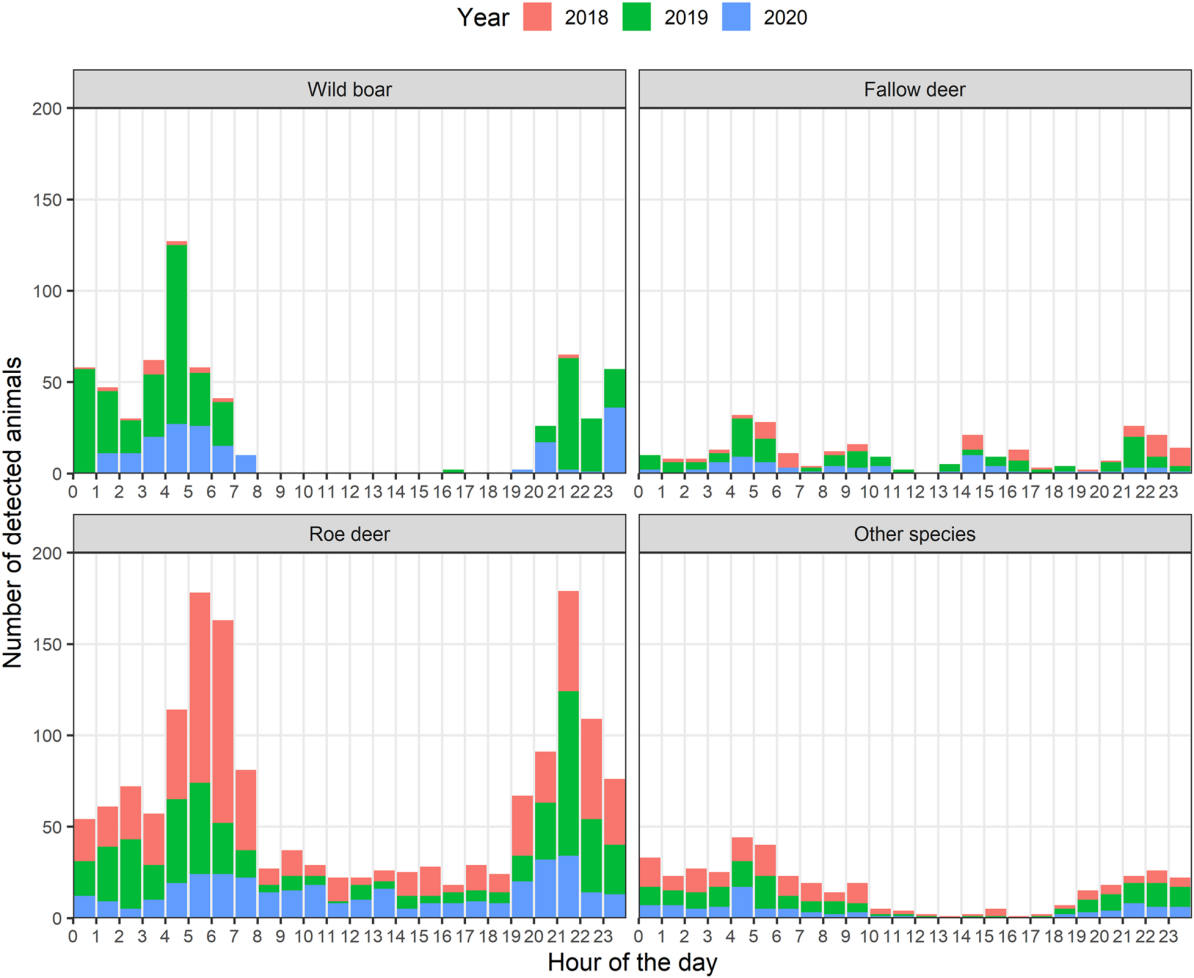
Hour histogram of all recorded animals in a day period.

Unlike people, animals were observed in the vast majority of cases during night hours or during sunrise or sunset. In the case of wild boar, 240 out of total 615 records (39.0%) appeared during the day (4 AM to 8 PM), which accounted for a statistically significant difference (Chi-squared = 29.634, df = 1, *p* < 0.001). Only 4 records of wild boar were recorded between 8 AM and 8 PM. The low number of wild boar records in 2018 is caused by previous ASF outbreak (in area was found 208 ASF positive wild boar carcasses^44^). However, the times of visits were in all 3 years comparable with morning and evening peeks regardless of population density. In the case of roe deer, 890 out of a total of 1589 records (56.0%) were detected during the day, although only 287 records of roe deer (18.1%) appeared between 8 AM and 7 PM. Other animal species (both martens, red fox, European badger or European hare) were detected during the day or night with similar frequency (203 records during the day from total of 400 records), although their activity was relatively low between 10 AM and 6 PM. Fallow deer were recorded during the whole day, with small peaks around 5 AM, 9 AM, 2 PM and 9 PM. The distribution of animal species records during the day is depicted in Fig. 4.

The testing for differences in frequency of animal visits between the people-influenced time (24 h after people visit) and the non-influenced time showed significant results (Wilcoxon paired-sample rank-sum test, V = 91, *p* < 0.001). The mean frequency of animal visits during the people-influenced time was 1.41 visits per day (95% CI 0.94–1.88), during the non-influenced time 3.76 visits per day (95% CI 2.79–4.73), which is ca. 160% higher.

The logistic regression model for predicting the presence of people on a particular camera trap record for each day, based on forest type and distance from roads and pathways, showed significant results for coniferous forests (pine/spruce), species-rich mixed forests and distances from roads and pathways. People were more likely to be recorded in pine/spruce forests (Effect coefficient = 1.9890), species-rich mixed forests (0.6703), and also far from roads (− 0.0025) and pathways (− 0.066). In all cases of statistically significant effects, very low *p* values (*p* < 0.001 in all cases) were observed. The results of the model are shown in Table1.

**Table 1:** Presence/absence probability of people on camera traps (daily data). The significant effects of measured parameters (*p* < 0.05) are marked in bold.

		Coefficient	Test. statistics	*p *value
	Intercept	− 3.2246	− 8.367	< 0.001
	Distance from roads	− 0.0025	− 3.525	** < 0.001**
	Distance from pathways	− 0.0066	− 4.683	** < 0.001**
Forest type	Pine/hornbeam	− 0.2150	− 0.413	0.68
	Pine/spruce	1.9890	3.842	** < 0.001**
	Oak/hornbeam	0.7403	1.519	0.13
	Hornbeam	− 0.4261	− 0.522	0.60
	Spruce	0.2420	0.573	0.57
	Species-rich mixed forest	0.6703	1.625	** < 0.001**

## Discussion

An unexpected effect of the COVID-19 pandemic was a surprisingly dramatic change in nature visits after the first measures reducing human mobility were implemented^27,45^. In countries where strict lockdown restrictions were imposed, a reduction in visitor numbers was initially observed^45,46^. Similarly, the number of visitors increased rapidly as soon as these restrictions were eased^45,46^. This pattern was also observed in our study, as the camera traps registered an increase of visitors of 151% during the COVID-19 period in 2020 compared to the same time period (May and June) in the previous year. This unprecedented boom of human visitors after short-term measures to mitigate the COVID-19 pandemic during the spring and summer 2020 were also reported from Nature Protected Areas and peri-urban forests globally^42,45,46^. For instance, the increase of forest visitors in peri-urban forests south of the Cologne-Bonn agglomeration (Germany) had increased almost 140% compared to pre-COVID-19 pandemic ^46^. A rapid increase was also observed in Oslo (Norway), where outdoor recreational activity increased by 291% during the 2020 lockdown dates relative to the 3-yr baseline average in previous years. In this case, the dramatic increase could be explained by easy availability of peri-urban forest around Oslo^47^.

In our study, the number of forest visitors could not only be compared to the pre-COVID-19 time period in 2019, but also to 2018, when a strict ban on entering the monitored area was applied due to an ASF breakout. This total restriction of forest access was relatively respected by women (only 6.7%) compared to men (93.3%). However, men and women accounted for 53.6% and 46.4% of the total number of visitors in 2019, and 56.7% and 43.3% in 2020. The same gender share was found in the normal situation (excluding ASF and COVID-19) in 2016 in the urban forests of Hradec Králové (56.2% men and 43.8% women; ^48^). In our study, the number of visitors during the period of forest entrance prohibition in 2018 was ca. 36% of the normal situation in 2019, and ca. 24% of the increased number of visitors in 2020. Therefore, the evaluation of entrance ban effectiveness in the ASF infected area is quite disputable. The complete standstill of any activities in the infected area was also prescribed e.g. in Belgium^49^, however the effectiveness was not evaluated. All activities outside of the forestry trails are particularly disturbing to wild boars. Indeed, whatever the activity being performed, the strict respect of trails is of prime importance^40^. The ASF spreading is affected by several factors such as natural movement of wild boar, including both home-range movement and long-range dispersal^37,39^. Therefore, the prohibition of human leisure activities is recommended in the outbreak area^40^.

In the normal situation, the number of daily visits is generally higher on weekends (up to 125%) compared to weekdays. This trend was confirmed e.g. in the peri-urban forest in the vicinity of Hradec Králové (CZ)^48^. In our study, we have also observed a relatively larger number of people on Saturdays, Sundays, and public holidays as expected, but surprisingly, the highest number of people were detected on Thursday, June 25, 2020. During the ongoing COVID-19 pandemic (2020), Thursdays were the days with the highest total number of recorded people, followed by Wednesdays and Sundays including public holidays. In 2019, 67% of visits were recorded during working days, increasing to 76% in 2020. This increase could be partly explained by the post-lockdown period, which increased the enthusiasm for going for a walk even on workdays. A comparable trend was described by Derks et al. (2020), where the clear difference between the number of visitors on weekdays and weekends substantially decreased after the lockdown measures were implemented, compared to the pre-COVID-19 times. In the COVID-19 period, people had more available time, more flexibility, more pressure at home, but also fewer alternative pastimes. In terms of the number of forest visitors, the tree species composition was an important predictor^48,50^. In our study, people preferred mixed coniferous forests (pine/spruce) and species-rich mixed forests, while conversely, attendance was the lowest in monospecific deciduous forests. Similarly, visitors to the Municipal forest in Ostrava (CZ) preferred more structured mixed forests, while spruce and pine were the most favorable tree species^51^. Italian responders preferred mixed forests (66%) over coniferous forests (28%) and deciduous forests (6%)^52^, while another study from the Protected Landscape Area Žďárské vrchy (CZ) documented the visitor preference of coniferous forests^53^.

The increase of outdoor recreational activities and nature tourism was indicated in a majority of published studies measured by Google mobility data, automatised visitor counters on forest roads, or by questionnaires ^46,47,48^. Therefore, human movement in the forests, in the context of wildlife disruption (outside of the forest roads), was impossible to evaluate. Conversely, in our study, the animals were monitored together with humans. The threat risk predicts that animals will avoid human disruption and react as expected during an encounter with a predator^54^, which was also confirmed in our study. The continuous monitoring showed that the presence of wildlife was significantly lower (more than 2.6 times) directly after human movement was recorded by camera traps—compared to the period without human presence. It is evident from these results, that on a short-time scale, the wildlife species avoided the areas visited by humans.

The impact of human activities can trigger behavioural and stress response in wild animals^55,56^, with a significant impact on entire wildlife populations^54,57^. When animals face the risk of predation, many engage in behaviour that reduces that risk, but also generate an increased energy cost^58^. For instance, animals may be more vigilant in the vicinity of different types of anthropogenic disturbances^54^, which results in a reduced immune response^59^, increased susceptibility to diseases^60,61^, reduced growth^62^ and a decreased fitness^63^. Moreover, the short-term disturbance could compel the animals to utilize lower quality habitats, which leads to damage of forest ecosystems or agricultural crops. The direct effect of human disruption has been proven, e.g. the increase of browsing pressure by ungulates^64,65^, which significantly affects the forest stability, wood production and sensitivity to fluctuation of climatic factors^66,67^. Another serious effect of human disturbance to wildlife is increased risk of wildlife-vehicle collisions in areas with higher human activity^68^ or close to urban areas with growing rate of human disturbances^69^.

However, the wildlife species that regularly encounter humans without negative consequences should get used to their presence^70,71^. The animals detected in our study area are mainly the common wild ungulates which are relatively accustomed to human disruption in the long-term, and able to occupy a highly modified cultural landscape. The tolerance is reflected e.g. by the dramatically increased population densities in Europe^72–74^. Conversely, the endangered animal species are much more sensitive to human disturbance than common species that successfully adapted to the recent land use changes. The most sensitive birds and mammal species decline or disappear from the highly disturbed sites, and the species composition shifts from “wild” species to cultural and human-associated species^5^. For instance, the study from northern Finland reveals that the proportion of ground-nesting birds is higher in forests than in tourist destinations^5^. Although open-cup nesters nesting on the ground showed a negative response to the number of visits on hiking trails, yet the species richness remained unaffected, as sensitive species were apparently replaced by generalist species^5,75^. However, the endangered species could successfully adapt to a regular and managed human presence in the vicinity of the areas of occurrence (e.g. human movement only of forest roads). A study from Germany (Lower Saxony) confirmed adaptation of black grouse (*Lyrurus tetrix*), critically endangered in Central Europe, to the human disruption. Differences in black grouse movement were monitored by GPS transmitters. Tagged individuals avoided the vicinity of public routes, and the contact distances were directly related to the intensity of human activity. Individuals used the vicinity of public trails at night and dawn but avoided these habitats during peak human activity around noon and afternoon^76^.

To our knowledge, this is the first study which compares the unique situation of differences in human visits in forests outside of forest roads in relation to distinct movement patterns. We have confirmed the rapid increase of visitors in the forests during the COVID-19 pandemic in May and June 2020. Our study confirmed that forest ecosystems play an indispensable role during pandemics and related economic crises—in relation to recreation services that the forests offer. On the other hand, apart from improving physical and mental health of visitors, they may possibly contribute to spreading of diseases. Contrarily, we have observed the decrease of visitors in the same area during the African swine fever outbreak, during which the entrance into the forests of the affected areas was completely prohibited. However, our findings demonstrate that the restrictions during the ASF outbreak were not fully respected. The trends in the rapid increase of human visitors outside of forest roads has a significant impact on wildlife behaviour. The places visited by humans were then avoided by animals in a short-time period. The confirmed impact of human visitation on wildlife behaviour highlights the need for new measures that should mitigate the negative effects on wildlife disruption through visitor's education, especially during the breeding season. Our results suggest that management measures are highly important, particularly in the areas where the threatened animal species live because they are much more sensitive to human disruption. Essentially, the political authorities should improve the communication of the restrictions concerning the African swine fever outbreaks which are expanding into new territories in central Europe. The disregard of entrance prohibitions could have an immeasurable impact on the disease transmission outside of the affected areas. Therefore, comprehension of the direct effects of human behaviour on wildlife and natural processes in forest ecosystems is indisputably important.

## Material and methods

### Study area

The study of human visits to the forest areas was focused on a location northeast of the city of Zlín in eastern Czech Republic (N 49°15′, E 17°44′). Zlín is the eleventh largest city in the Czech Republic with 74,921 residents. The population density of the Zlín district is 187 inhabitants/km^2^, while 72.5% inhabitants live in towns. The proportion of women to men is 51.5% to 48.5%, and the average age is 39.9 years (www.zlin.eu). The location was selected due to the African swine fever outbreak which began on June 21, 2017. The outbreak was officially eradicated—according to the European commission—on March 12, 2019.

The average annual temperature in the location reaches 13.2 °C, and the total sum of precipitation is 656 mm, while it is 20.5 °C and a monthly 73 mm in the studied months. The study area has a humid continental climate characterized by hot and humid summers and cold to severely cold winters (Cfb region) according to the world-wide^77^ classification.

The acreage of the monitored area was about 5430.6 ha with the following land use composition: standard commercially managed forests—26.6% (1443.8 ha), human settlements—1.7% (92.5 ha), agricultural land—71.6% (3889.7 ha), and water bodies—0.1% (4.6 ha). Thirteen villages are located in the area of interest, with an average population of 863 inhabitants. The altitude ranges from 221 to 413 m a.s.l.

### Study design and camera trap monitoring

**Figure 5:**
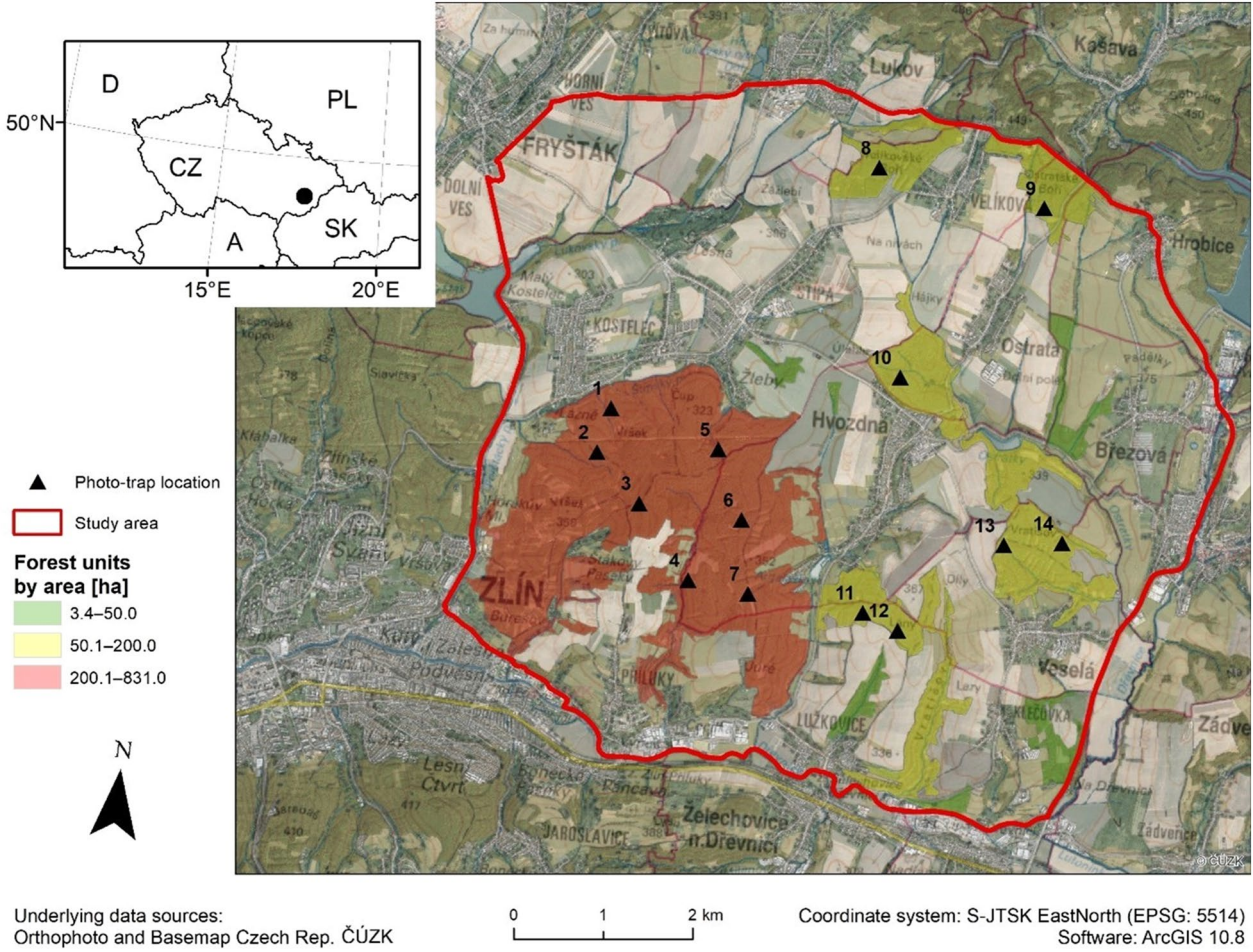
Distribution of forest areas and camera traps in the study area (▲). The study area (northeast from the city of Zlín) is marked by a black dot (●) for context of the Czech Republic and neighboring countries. This map was created in ESRI ArcMap 10.8 (https://desktop.arcgis.com/zh-cn/arcmap/).

The forest areas in the location of interest were sorted according to their acreage into categories of small forests (0–50 ha; marked by green), bigger forests (50.1–200 ha; yellow) and large forest complexes (˃200 ha; red). Human visits were monitored only in the forests larger than 50 ha (Fig. 5.). Human presence in the forest and the activity of common animal species was monitored by 14 randomly placed camera traps, i.e. 1 camera trap per 100 ha of forest area. The locations for camera traps were previously selected via random points tool in GIS software (ArcGIS 10.8). All camera traps were placed at least 100 m from the forest edge and forest road. The monitoring was implemented in three consecutive seasons (2018, 2019 and 2020) utilizing the same time period from 1^st^ May to 30^th^ June (61 monitoring days every year).

The UO Vision UV 595 HD cameras were used with the following parameters: invisible IR camera (resolution of 12 megapixels), utilizing a trigger speed of 0.65 s and HD video (1080P) recording (for more information see www.uovision.com). All cameras were installed on a tree at a height between 1 and 1.5 m. The date and time were recorded automatically at the beginning of each video. The sites were inspected every 3 weeks to check the cameras and download recorded videos. The camera traps triggered video recording automatically when motion was detected. Settings of the camera traps were set to 30-s videos with a 1-min window between each video sequence.

### African swine fever pattern in 2018

Visiting the affected location was banned during the time of African swine fever to prevent possible transmission of the disease out of the outbreak area. These restrictions were valid from August 9, 2017, to November 26, 2018. Regulation forbade the entry into the suburbs of municipalities in the defined area, with the exception of gamekeepers exercising measures to prevent the spread of African swine fever, and with the exception of individuals permitted by the Mayor of the city of Zlín. The area was intensively monitored by police patrol, with the fines for infringement of the regulations reaching 20,000 CZK (approx. 770 EUR).

### Standard situation in 2019

The movement of citizens in the forests of the Czech Republic is defined by the Article 19, Act No. 289/1995 Coll. In general, every individual is entitled to enter forest stands at their own risk. Visitors must not damage the forest, nor interfere with the forest environment. They are obliged to follow the instructions of the owner or tenant of the forest and their staff. Act No. 289/1995 also further defines restrictions for forest users.

### COVID-19 pandemic in 2020

On March 12 at 2.00 PM, a state of emergency was declared pursuant to Articles 5 and 6 of the Constitution Act No. 110/1998 (on the Security of the Czech Republic) limiting certain rights and freedoms of citizens (Resolution No. 194/2020). On March 13, the security measures progressed further, as full-time education and organized indoor and outdoor events and activities of more than 30 people were banned, and facilities such as gyms, wellness centers and solariums were closed (Resolution No. 72/2020, Resolution No. 84/2020). With effect from March 14, 2020, the government banned all foreign entry into the territory of the Czech Republic from high-risk areas, with specified exceptions (Resolution No. 76/2020). From March 16, 0:00 AM, until March 24, 06:00 AM, the free movement of citizens was limited throughout the Czech Republic with several exceptions, such as visits to nature and parks. Furthermore, the government strongly recommended to all employers to implement and encourage home office in order to limit the movement and direct contact of citizens^78^ (Resolution No. 85/2020). The state of emergency was further extended until May 17, which included most of the previously declared restrictions (Regulation No. 219/2020). However, the restrictions regarding free movement and outdoor activities were gradually lifted starting on April 7, when individual outdoor sports were permitted, and on April 24, free movement of the public was restored (in groups smaller than 10 people). On May 11, full-time education was restored for the last year of primary schoo ls and the last year of secondary schools (Regulation No. 220/2020), all businesses including shops, shopping centers, and gyms were re opened on May 25, and on June 8, all pupils were allowed to return to schools^79^.

### Statistical analysis

All the videorecords were manually inspected. Records were sorted for statistical analysis and the database was created in MS Excel according to year, date, time of visit, wildlife species, and the visitors data. The visitors data set was divided according to age (subadults, adults and seniors) and gender (male and female). The possible duplicity records of visitors were not included in the analysis. The animal data set was divided according to wildlife species present in the monitored area to wild boar (*Sus scrofa* L.), fallow deer (*Dama dama* L.), roe deer (*Capreolus capreolus* L.), and a group of other animal species, including martens (pine marten [*Martes martes* L.] and stone marten [*Martes foina* L.]), red fox (*Vulpes vulpes* L.), European badger (*Meles meles* L.), and European hare (*Lepus europaeus* Pallas).

For a basic overview on the record of people visiting in different years, a simple histogram of human records divided into subadults, adults and seniors is presented. We have also presented basic statistics and comparisons of selected study years. The upward trend of human records between study years was confirmed by linear regression. For the description of most frequent weekdays, histograms for each study year were presented. We have tested for differences in human records on different weekdays together for all study years via chi-squared test. The testing for differences in proportion to visits on different weekdays in 2019 and 2020 were also tested via chi-squared test.

The times of human and animal visits are presented by an hour histogram of records for each study year. In the case of wild boar (the game species with the most prominent differences in the number of records during the day vs. the number of night records), the differences in proportion of day and night records were also tested by chi-square test (according to accurate sunrise and sunset in particular days; Central European Time).

We have tested for differences in animal visit frequency during “people-influenced” time (24 h after the time when people were detected on camera traps) and non-influenced time (opposite cases, e.g. more than 24 h from the last people detected). The intensity was calculated as a sum of detected animals (number of records) divided by the total “people-influenced” time, and vice versa for each camera trap in all study years combined.

A logistic regression model was used to test the dependency of people’s presence, which were recorded by camera traps, at a distance from the roads and pathways, and the forest type involved—Scots pine (*Pinus sylvestris* L.) and European hornbeam (*Carpinus betulus* L.) forests, pine and Norway spruce [*Picea abies* (L.) Karst] forests, oak (*Quercus* spp.) and hornbeam forest, monospecific hornbeam forests, monospecific spruce forests, and species-rich mixed forests, which were represented in the data collection.

All computations were performed in R software on selected alpha level of 0.05. The plots were made in “ggplot2”package^80^.
